# Patient injuries after total hip arthroplasty for osteoarthritis in Sweden over 10 years: a cohort study based on 1,343 patients

**DOI:** 10.2340/17453674.2026.45971

**Published:** 2026-06-04

**Authors:** Siri HEIJBEL, Sabina RAHMANIAN, Pelle GUSTAFSON, Harald BRISMAR, Annette W-DAHL, Margareta HEDSTRÖM

**Affiliations:** 1 Department of Clinical Science, Intervention and Technology (CLINTEC), Karolinska Institutet, Stockholm; 2 Medical Research Internship, Karolinska University Hospital, Stockholm; 3 Department of Orthopedics, Clinical Science Lund, Lund University, Lund; 4 The National Swedish Patient Insurance Company (Löf), Stockholm; 5 Trauma and Reparative Medicine Theme (TRM), Karolinska University Hospital, Stockholm; 6 The Swedish Arthroplasty Register, Gothenburg, Sweden

## Abstract

**Background and purpose:**

Injuries after primary total hip arthroplasty (THA) can be compensated through the National Swedish Patient Insurance Company (Löf). This study examined injury incidence, types, consequences, and subgroup differences in compensated claims following primary THA for osteoarthritis (OA) in Sweden over 10 years.

**Methods:**

Data from Löf and the Swedish Arthroplasty Register were used for compensated claims following primary THA between 2012 and 2021. Injury incidence and subgroup differences by sex, age, OA diagnosis, and surgical volume were analyzed. Injury types, severity, reoperation rate, and surgical approach were evaluated. Absolute risk differences (ARD) and incidence rate ratios (IRR) were calculated.

**Results:**

1,343 injuries were followed up. The injury incidence was 1.3%. Injury rates were higher in patients < 65 years than ≥ 65 years (ARD 0.2%, 95% confidence interval [CI] 0.1–0.3), in OA due to hip dysplasia compared with primary OA (IRR 1.78, CI 1.31–2.35), and at low- and medium-volume compared with high-volume hospitals (IRR 1.35, CI 1.11–1.64; IRR 1.27, CI 1.14–1.42). In surgery-related injuries (n = 1,251), infection (38%, CI 35–41) and nerve injury (22%, CI 20–25) were the most common injury types, and direct lateral incisions were more frequent than national reference proportions of all THAs during the study period (ARD 9%, CI 6–12). Permanent disability occurred in 74% (CI 72–77) of the claims, and the 2-year reoperation rate was 51% (CI 48–54) (0.4% of the total population).

**Conclusion:**

The injury incidence after primary THA was 1.3% and frequently resulted in permanent disability and reoperation. Infection and nerve injury were the most common injury types. Higher injury rates in younger patients, hip dysplasia, and lower-volume hospitals highlight potential areas for prevention and increased awareness.

Previous studies in other Scandinavian countries report incidence rates of compensated patient injury claims after total hip arthroplasty (THA) that range from 0.8% to 2.5% [1-3]. An earlier Swedish study, based on data from 1997 to 2004, reported an incidence ranging between 0.62% and 1.00%, depending on the fixation method [4]. However, the types of injuries were not reported, leaving the distribution unknown. Data from the Swedish Arthroplasty Register (SAR) shows that infections, hip dislocations, and periprosthetic fractures are the most common causes of reoperation within 2 years after primary THA [5].

In Sweden, patient injury claims are managed by the Swedish Patient Insurance Company (Löf), the national statutory patient insurance provider for publicly funded healthcare [6]. Although compensated claims are injuries considered avoidable, knowledge of injury types and subgroup differences following primary THA remains limited. Consequences of patient injuries can include long-term patient suffering, increased healthcare utilization, and substantial costs. Given the high volume of THAs performed, improving understanding of these injuries may support patient safety initiatives and help reduce preventable harm.

Our primary aim was to examine the injury incidence and describe injury types of compensated claims to Löf after primary THA for osteoarthritis (OA) in 2012–2021. Secondary aims included subgroup investigations by sex, age, OA diagnosis, hospital surgical volume, reoperation rate, incision type, and fixation method.

## Methods

### Study design and data sources

The STROBE statement guidelines were followed. This retrospective cohort study used data from Löf and the SAR. Claim assessments are regulated by Swedish law, specifically the Patient Injury Act (SFS 1996:799). When a patient injury is suspected or certain, healthcare providers are legally obligated to inform patients of the possibility of filing a claim with Löf. Compensation is granted for financial loss and permanent functional disability from injuries deemed avoidable. It is provided if it results from injuries during examinations or treatments, accidents during medical care, faulty medical equipment, incorrect or delayed diagnosis, preventable infections, or medication errors. Injuries in the Löf database are classified using the Swedish version of the International Statistical Classification of Diseases and Related Health Problems (ICD-10-SE). Time limits for filing claims changed in 2015. For injuries before 2015, claims had to be filed within 3 years of awareness and no later than 10 years after the injury. Since 2015, claims have been allowed within 10 years of awareness. In terms of surgery-related injuries after THA, Löf grants compensation to all hospital-acquired infections debuting within 12 months, early aseptic loosening (typically within 2 years), severe nerve injuries, leg length discrepancies > 2 cm during 2012–2015 and > 1.5 cm from 2016, all perioperative fractures, and other avoidable injuries due to substandard surgical performance. Decisions regarding patient injury claims related to orthopedic injuries are supported by orthopedic surgeons, and specialists in other relevant medical specialties support decisions for other types of injuries.

### Data collection and analyses

**Figure 1:**
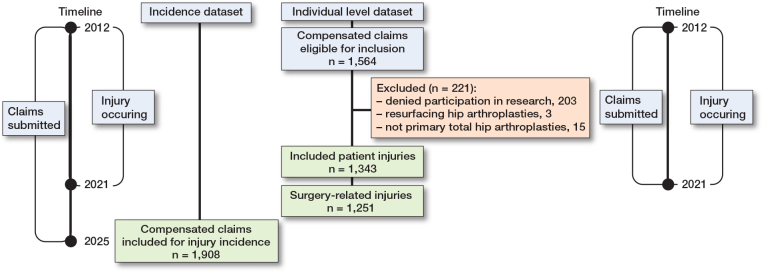
Flow diagram of the study showing details for the exclusion and inclusion of compensated claims for the incidence dataset and the individual level dataset.

For the incidence dataset, aggregated data from all compensated claims occurring between January 1, 2012 and December 31, 2021, submitted until December 31, 2025, following primary THA for OA (ICD-10-SE codes M16.0–M16.7 and M16.9) and procedure coding (KVÅ) NFB29/39/49 was collected to estimate the overall and annual injury incidence (Figure 1). The overall injury incidence was calculated as the number of compensated claims divided by the total number of primary THAs reported to SAR during the period.

Data for the individual level dataset used for detailed analyses and review was collected from compensated claims submitted and occurring between January 1, 2012 and December 31, 2021 (see Figure 1). Claims were excluded if they involved resurfacing THA, were incorrectly labeled as primary but occurred during reoperation or refused participation in research.

Medical records and claim decision reports from Löf were reviewed to determine injury type and severity, as well as patient characteristics, including sex, age at injury, hospital of primary surgery, injury code, surgery date, and surgical incision. Injury severity was defined as compensation for temporary pain and suffering or economic loss, and permanent medical disability (1–15% or ≥ 16%), or death. National and hospital-level data on the number of primary THAs, reoperations, and implant fixation methods was obtained from the SAR. Medical records from Löf were used to assess reoperations if they were not registered in the SAR. The completeness of primary THA during the study period in the SAR was 97–99%, and 92–94% of revision surgeries. The completeness of other reoperations was less certain [5].

### Differences by sex, age, and OA diagnosis

Injury rates and differences by sex, age (< 65 years and ≥ 65 years), and OA diagnosis were calculated based on 1,343 compensated claims (see Figure 1). Subgroup differences were calculated as the proportion of compensated claims within each group relative to the total number of primary THAs in that group reported to the SAR.

Hospitals were stratified by average annual THA volume into low-volume (< 100 THAs/year), medium-volume (100–300 THAs/year), and high-volume (> 300 THAs/year) categories, based on SAR data during the study period. The rate of compensated claims by hospital surgical volume was based on 1,536 compensated claims (see Figure 1). Hospital-specific data for 10 claims was missing in Löf and could not be retrieved from the SAR or medical records due to a lack of patient-specific identifiers.

### Injury type

A detailed evaluation was performed using medical records and claim decision reports. Injuries that involved the surgical site or the operated-on joint were defined as surgery-related, while other injuries were defined as other treatment-related injuries. When patients had multiple injuries, each was considered separately if independently linked to the primary surgery. For example, a hip dislocation due to component malposition and a periprosthetic infection were counted as 2 injuries. However, when 1 injury caused another, only the first was recorded, for example, an acetabular perioperative fracture resulting in cup loosening. Surgery-related injuries were grouped into the following categories: infection, nerve injury, gluteal insufficiency, leg length discrepancy, perioperative fracture, hip dislocation, and aseptic loosening. Less frequent injuries attributed to suboptimal surgical performance were grouped as “substandard surgery.”

### Surgical factors

Information on incision type (posterior, direct lateral in lateral or supine position, or other) was collected from Löf medical records. Data on implant fixation method (cemented, uncemented, or hybrid) was obtained from the SAR. For cases with hybrid fixation, medical records from Löf were reviewed to determine whether the cup or stem was cemented. The distribution of patient injuries by incision type and fixation method was compared with the national mean proportions reported in the SAR during the study period. We assumed that the choice of fixation method and incision type was independent.

### Reoperations

Patients with compensated claims were linked to the SAR to assess the proportion who sustained surgery-related injuries and underwent reoperation within 2 years following their primary THA. The reoperation rate between injury types was compared. A reoperation was defined as any open surgical procedure related to the primary THA. This included revision surgeries, in which part or all of the implant was exchanged, added, or removed, as well as other open procedures that did not involve implant interaction.

### Statistics

Age was presented as the mean and range. Sex was presented as proportions and numbers. Comparisons between observed proportions in the study cohort and national reference proportions were estimated using absolute risk differences (ARD) with 95% confidence intervals (CI). The ARD was calculated from raw data based on the observed difference in proportions between the groups. The corresponding CI was estimated using the standard error of the difference in proportions. Differences in injury rates by OA diagnosis and hospital surgical volume, and subgroup differences in injury types, were analyzed using Poisson regression. Results were presented as incidence rate ratios (IRRs) with primary OA, high-volume hospitals, and infections as the reference groups, respectively. Poisson regression allows estimation of the relative rate for a rare outcome and facilitates interpretation, as it is approximately equal to relative risk.

Missing outcome data was 1.4% for incision type, 0.2% for fixation method, and 0.7% for hospital site. Analyses were conducted using complete cases. The study includes multiple outcomes and subgroup comparisons. The analyses were descriptive and aimed to assess patterns of patient injuries and associated factors rather than to formally test predefined hypotheses. Therefore, no adjustment for multiple comparisons was applied. Instead, CIs are presented to estimate the uncertainty of observed differences [7].

### Ethics, funding, use of AI tools, data sharing plan, and disclosures

The study was approved by the Swedish Ethical Review Authority, 2022-06766-01. The study was partially funded by the Karolinska Institutet research internship. ChatGPT 4o was used to rephrase individual sentences, and Microsoft Copilot for proofreading. The individual-level data is protected by Swedish law but can be accessed after receiving approval from the Swedish Ethical Review Board. Complete disclosure of interest forms according to ICMJE are available on the article page, doi: 10.2340/17453674.2026.45971

## Results

144,848 THAs were performed between 2012 and 2021, and 1,908 compensated claims from injuries during this period were available for incidence estimation (Figure 1). Furthermore, for detailed analysis and review, 1,546 compensated claims were eligible; 1,343 were included in the analysis, of which 1,251 were surgery-related (Figure 1).

### Injury incidence and differences across years, sex, age, type of OA, and hospital surgical volume

**Figure 2:**
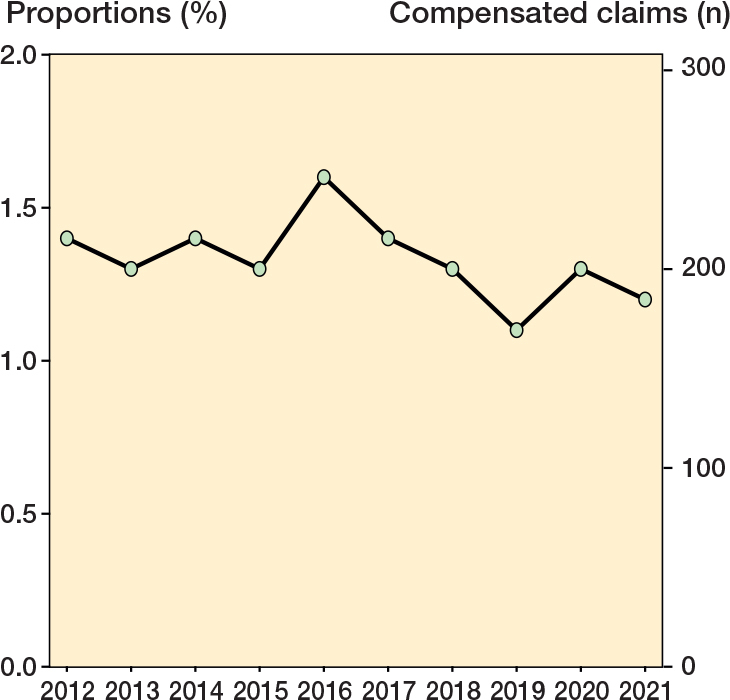
Proportions (%) and numbers of compensated claims per year 2012–2021.

The injury incidence was 1.3%, varying from 1.1% in 2019 to 1.6% in 2016 (Figure 2). The median time from injury to filing a claim was 6 months (range 0–112 months), with 70% of claims filed within the first year after the injury. Patients with compensated claims had a mean age of 67 years (range 24–93), and 57% were female. For all primary THAs performed during the study period, the mean age was 69 years (range 14–98), and 57% were female. Patients < 65 had a higher rate of compensated claims (1.1%) than those ≥ 65 (0.9%) (ARD 0.2%, CI 0.1–0.3). The rate did not differ between females (0.9%) and males (0.9%), ARD 0% (CI –0.01 to 0.01). Compared with primary OA (0.9%), the rate of compensated claims was higher in patients with OA secondary to hip dysplasia (1.6%), IRR 1.78 (CI 1.31–2.35) (Table 1). Furthermore, compared with high-volume hospitals (0.9%), the rate of compensated claims was higher in low-volume hospitals (1.3%), IRR 1.35 (CI 1.11–1.64), and medium-volume hospitals (1.2%), IRR 1.27 (CI 1.14–1.42).

**Table 1:** Number and rate of compensated claims subdivided by diagnosis

OA diagnosis	Total number of THAs	Patients with compensated claims, n (%)	IRR (CI)
Primary OA (M16.0, M16.1, M16.9)	138,998	1,272 (0.9)	ref
OA secondary to hip dysplasia (M16.2, M16.3)	2,888	47 (1.6)	1.78 (1.31–2.35)
Post-traumatic OA (M16.4, M16.5)	1,276	9 (0.7)	0.91 (0.51–1.47)
Other secondary OA (M16.6, M16.7)	1,686	14 (0.8)	0.77 (0.37–1.39)

Abbreviations: OA = osteoarthritis; THA = total hip arthroplasty; ref = reference category; IRR = incidence rate ratio; CI = 95% confidence interval.

### Consequences for the patients

Most patients sustained a permanent disability from their injury of either 1–15% in 72% (CI 70–75), or ≥ 16% in 2.2% (CI 1.5–3.2). 25% (CI 23–28) were not permanently disabled but received compensation for temporary pain and suffering or economic loss. Compensation for death was granted in 3 patients, 0.2% (CI 0.04–0.70): 1 from a massive intraoperative bleeding, 1 from deterioration by repeated surgery due to prosthetic joint infection, and 1 from delayed diagnosis of myocardial infarction.

### Injury types

Among the reviewed compensated claims, 1,368 injuries were identified in 1,343 patients, of which 1,251 (91%) were surgery-related (see Figure 1). The most common surgery-related injury was infection (38%, CI 35–41; n = 473), predominantly deep joint infections, with a small proportion (1%) being superficial wound infections. This was followed by nerve injury (22%, CI 20–25; n = 279), gluteal insufficiency (8%, CI 7–10; n = 102), leg length discrepancy (8%, CI 6–9; n = 94), perioperative fracture (7%, CI 6–9; n = 92), hip dislocation (7%, CI 6–8; n = 85), substandard surgery (6%, CI 5–7; n = 74), and aseptic loosening (4%, CI 3–5; n = 52). Within 2 years of primary surgery, 51% (CI 48–54; n = 638) of surgery-related injuries required reoperation (0.4% of the total population). Less frequent injuries, grouped as substandard surgery, are summarized in Table S2 (see Supplementary data). Additionally, 117 other treatment-related injuries were identified and are listed in Table S3 (see Supplementary data).

### Differences in sex, age, and reoperation rate depending on injury type

Compared with the reference category infection, females were overrepresented in most injury types, particularly gluteal insufficiency IRR 2.24 (CI 1.71–2.90) and nerve injury IRR 2.18 (CI 1.79–2.67) (Table 2). The reoperation rate varied across injury types (Table 2). Age differences were less consistent. Among patients < 65 years old, gluteal insufficiency was less common, IRR 0.5 (CI 0.32–0.78), whereas aseptic loosening tended to be more frequent, IRR 1.48 (1.00–2.13) (Table 2).

**Table 2:** Differences in sex, age, and reoperation rate depending on injury types. Values are incidence rate ratio with (95% confidence interval)

Injury type	Reoperation	Females	Age < 65
Infection	Ref	Ref	Ref
Nerve injury	0.03 (0.01–0.05)	2.18 (1.79–2.67)	0.84 (0.65–1.07)
Gluteal insufficiency	0.25 (0.16–0.38)	2.24 (1.71–2.90)	0.51 (0.32–0.78)
Leg length discrepancy	0.09 (0.04–0.17)	1.92 (1.44–2.55)	0.95 (0.66–1.34)
Perioperative fracture	0.39 (0.27–0.54)	2.18 (1.64–2.86)	0.97 (0.67–1.37)
Hip dislocation	0.84 (0.64–1.08)	1.47 (1.05–2.03)	1.17 (0.82–1.63)
Substandard surgery	0.45 (0.31–0.64)	1.80 (1.30–2.46)	0.84 (0.54–1.25)
Aseptic loosening	0.69 (0.47–0.96)	1.34 (0.86–2.00)	1.48 (1.00–2.13)

### Nerve injuries

Of all nerve injuries, 91% (n = 254) involved the sciatic nerve, and 86% (n = 218) of these had peroneal nerve paralysis. Femoral nerve injuries accounted for 9% (n = 24), and 1 patient had an obturator nerve injury. In 57% (n = 159), the lesions were confirmed by neurophysiological testing, while the remaining injuries were assessed and compensated based on clinical symptoms described in the medical records. At the last follow-up, a minimum of 1 year after primary surgery, only 1% (n = 3) of nerve injuries had fully resolved.

### Type of incision

Of the surgery-related injuries, 46% (n = 576) occurred in patients operated on through a posterior incision, which was 10% (CI –13 to –7) less frequent than the national reference proportion during the study period (56%). Moreover, 52% (n = 653) were operated on through a direct lateral incision, which was 9% (CI 6–12) more frequent than the national reference proportion during the study period (43%). The remainder were other incisions. Accordingly, the distribution of incision types among patient injuries differed from the national reference proportion and varied across injury types (Table 3).

**Table 3:** Proportion (%) and absolute risk differences (ARD) (95% confidence interval)^a^ compared with all THAs during the study period (2012–2021) based on incision type and fixation method

Item	All THAs 2012–2021 (%)	Infection n = 473		Nerve injury n = 279		Gluteal insufficiency n = 102		Leg length discrepancy n = 94	
		(%)	ARD (CI)	(%)	ARD (CI)	(%)	ARD (CI)	(%)	ARD (CI)
Posterior incision	56	41	–15 (–26 to –4)	56	0 (–13 to 13)	6	–50 (–54 to –46)	44	–12 (–23 to –1)
Direct lateral incision	43	57	14 (10 to 19)	41	–2 (–8 to 4)	94	51 (47 to 56)	62	19 (9 to 29)
Cemented fixation	57	50	–7 (–12 to –3)	61	4 (–2 to 10)	80	23 (16 to 31)	59	2 (–9 to 12)
Uncemented fixation	27	37	10 (6 to 15)	20	–7 (–12 to –3)	13	–14 (–21 to –8)	17	–10 (–18 to –2)
Hybrid fixation	16	13	–3 (–6 to 0)	19	3 (–2 to 8)	7	–9 (–14 to –4)	25	9 (0 to 17)

a Negative values are lower proportions than the national reference; positive values are higher proportions than the national reference.

**Table 3:** Continued.

Item	All THAs 2012–2021 (%)	Perioperative fracture n = 92		Hip dislocation n = 85		Substandard surgery n = 74		Aseptic loosening n = 52	
		(%)	ARD (CI)	(%)	ARD (CI)	(%)	ARD (CI)	(%)	ARD (CI)
Posterior incision	56	61	5 (–8 to 18)	67	11 (–3 to 25)	53	3 (–15 to 9)	52	4 (–16 to 8)
Direct lateral incision	43	37	–6 (–16 to 4)	32	–11 (–21 to –1)	46	3 (–8 to 14)	46	3 (–1 to 17)
Cemented fixation	57	37	–20 (–30 to –10)	32	–25 (–35 to –15)	58	1 (–10 to 10)	31	–26 (–39 to –14)
Uncemented fixation	27	37	10 (0 to 20)	57	30 (20 to 40)	16	–11 (–19 to –2)	44	17 (4 to 31)
Hybrid fixation	16	26	10 (1 to 19)	12	–4 (–11 to 3)	23	7 (–3 to 17)	25	9 (–3 to 21)

### Implant fixation method

Among the compensated claims, 53% (n = 662) involved cemented fixation, which was 4% (CI –7 to –1) lower than the national reference proportion (57%). Furthermore, 30% (n = 377) involved uncemented fixation, which was 3% (CI 1–6) higher than the national reference proportion (27%), and 17% (n = 209) involved hybrid fixation, the same as the national reference proportion (17%). Data was missing in 3 patients due to aborted surgery (i.e., complication during surgery). The distribution of implant fixation methods among compensated claims varied across injury types (see Table 3).

## Discussion

We aimed to examine the injury rate, injury types, consequences, and subgroup differences among compensated claims following primary THA for OA in Sweden over 10 years. We found that the injury rate was 1.3% during 2012–2021. Three-fourths resulted in permanent functional disability. The rate of compensated claims was lower in high-volume hospitals and higher in younger patients and in patients with OA secondary to hip dysplasia. Infections and nerve injuries were the most common injury types. Patients operated on through direct lateral incisions were more commonly compensated than expected relative to national reference proportions.

The injury incidence was higher than previously reported for Löf claims from 1997–2004 [4]. The rate of compensated claims varied slightly over the study period. The injury rate was higher at low- and medium-volume hospitals than at high-volume hospitals, consistent with findings from Finland [3] and Norway [2], but in contrast to a recent Dutch study [8]. This may partly reflect that healthier patients are more often treated at high-volume hospitals, but previous studies have also associated low surgical volume with an increased risk of adverse events [9-13].

The present and previous studies suggest patient injuries are underreported [1,14,15]. Kasina et al. found that only 25% of prosthetic joint infections after THA in Sweden were filed as claims over 3 years [14]. In the present study, claims for prosthetic joint infections (0.35% of all THAs) were nearly 4 times lower than the reoperation rate for infections reported in SAR (1.2%) [5]. Most infections, except rare high-risk cases, are considered avoidable and eligible for Löf compensation. Underreporting may reflect limited awareness among healthcare providers and patients, as well as confusion between claims to Löf and malpractice investigations by regulatory authorities.

Younger patients were slightly overrepresented among compensated claims, consistent with findings from Finland [3]. This may reflect more complex cases such as OA secondary to hip dysplasia, which has also been shown to have a higher rate of compensated claims compared with other OA diagnoses in the present study. Furthermore, higher functional demands in younger patients could explain this, or a greater likelihood of seeking care, whereas older patients may be less likely to file claims [16]. No overall sex difference was observed compared with national reference proportions, aligning with recent Norwegian findings [2], though earlier Finnish data suggested males were less likely to file claims [16]. However, across injury types, compared with infections, females were overrepresented, particularly in gluteal insufficiencies and nerve injuries (see Table 2). This is consistent with previous claims data [3] but contrasting with a study reporting higher rates of infection and dislocation among females [17]. Half of the affected patients required reoperations within 2 years, compared with 2.2% of all THAs, according to the SAR [5]. Moreover, infections were the injury type requiring the most reoperations in the study cohort, while gluteal insufficiencies and nerve injuries were associated with a lower reoperation rate (see Table 2). These findings are expected, as prosthetic joint infections usually require at least irrigation and debridement. Overall, these findings suggest that injury patterns vary by age, type of OA, and sex.

Nerve injuries were the second most common injury type, mainly involving the sciatic nerve. These injuries mostly presented with symptoms from the peroneal portion, consistent with previous findings from Denmark [1]. This is likely due to the structure of the branches in the proximal part of the sciatic nerve, since the peroneal portion has fewer tightly packed fascicles with less connective tissue, making it more vulnerable to direct damage than the tibial portion [18]. Only 1% of patients with nerve injury showed complete recovery at follow-up, consistent with the slow rate of nerve regeneration. Also, patients with milder nerve injuries without lasting disability may not file claims.

Compared with national reference proportions, patient injuries were more frequent with a direct lateral incision than with a posterior incision, though the clinical significance cannot be determined from this study. Gluteal insufficiency occurred in 8% of the surgery-related injuries, mainly in patients operated on by direct lateral incision (see Table 3). A Norwegian study reported fewer gluteal patient injuries as the use of the direct lateral incision declined to below 5% of THAs [2]. Expectedly, dislocations had a disproportionately higher percentage of posterior incisions (see Table 3), although a Swedish register study showed a decline in reoperation due to dislocation associated with posterior incision and hypothesized an improved surgical technique explaining these results [19].

The fixation method was compared with national reference proportions and cemented implants predominated among gluteal insufficiencies, whereas uncemented or hybrid implants were more common in cases of infection, perioperative fractures, dislocations, and aseptic loosening. This aligns with previous Nordic reports [20] but differs from studies from the United States [21,22], perhaps reflecting differences in clinical practice. These variations underscore the influence of surgical approach and highlight the need for further research.

### Strengths

Data from Löf and the SAR are nationwide and cover all hospitals performing THA, with 98% completeness. A decade-long observation yields a relatively large study population despite the low proportion of injuries. A detailed review of medical records allowed an accurate assessment of injury types. This level of detail has previously been limited due to imprecise or vague diagnostic coding in aggregated data from Löf [23].

### Limitations

There was a potential underreporting of patient injuries, variability in reporting across injury types or hospitals, and data collection while patients could still file claims. The inter-rater reliability among medical experts is unknown, although assessments follow rule-based procedures. The specialist level was not included, which, however, may not have influenced the results as a recent study showed no difference in overall complications except infection [24].

Hospitals have not automatically received their Löf data on patient injuries since 2017 for legal and practical reasons. Although systematic feedback is lacking, hospitals can request their data, which may support self-monitoring and quality improvement. An earlier Finnish study suggested that, despite being rare, patient injury claims can serve as a complementary quality indicator [25].

### Conclusion

The injury incidence after primary THA in Sweden was 1.3%. 74% of affected patients sustained permanent disability, and 51% underwent reoperation. Infection and nerve injury were the most common injury types. Higher injury rates in younger patients, hip dysplasia, and lower-volume hospitals highlight variability, and increased awareness may support clinical decision-making and prevention strategies.

### Supplementary data

Tables S2 and S3 are available as Supplementary data on the article homepage, doi: 10.2340/17453674.2026.45971

## Supplementary Material



## Notes

Conception and design: MH, AWD. All authors contributed to finalizing the methodology. Data collection: SH, SR. Statistical analyses: SH. All authors contributed to the interpretation of the results, to drafting and revising the manuscript. All authors approved the final version.

Handling co-editors: Per Henrik Randsborg and Robin Christensen

*Acta* thanks Kim Lyngby Mikkelsen and Yrjänä Nietosvaara for help with peer review of this manuscript.
