# Supplementary material for: Patient injuries after total hip arthroplasty for osteoarthritis in Sweden over 10 years: a cohort study based on 1,343 patients
Source: Acta Orthop. 2026 Jun 4;97:329–35. doi: 10.2340/17453674.2026.45971 (PMC13234912; doi:10.2340/17453674.2026.45971)
Supplement: Supplementary file 1 [file ActaO-97-45971-s1.pdf]

Supplementary Table 2. Detailed types of the 74 injuries categorized as substandard surgery in patient injuries after THA 2012–2021

**Injury type**	**n (%)**
Unintentional retained bone cement or leakage	26 (35)
Mispositioned or misaligned component	25 (34)
Incorrect implant size	9 (12)
Bleeding [a]	5 (7)
Retained foreign bodies	4 (5)
Implant breakage due to metal fatigue	2 (3)
Other [b]	1 (1)
Component mismatch	1 (1)
Surgical wound rupture	1 (1)

[a] Bleeding leading to further complications such as reoperations, death, and prolonged hospital stay.

[b] Surgical failure leading to temporary Girdlestone during primary surgery and reoperation a month later.

Supplementary Table 3. Description of the 117 other treatment-related injuries after THA 2012-2021.

**Injury type**	**n (%)**
Nerve injury [a]	31 (26)
Wound [b]	24 (21)
Others	19 (16)
Anesthesia-related	13 (11)

Inpatient falls	9 (8)
Failed medical device	9 (8)
Urological injuries	8 (7)
Thromboembolism	4 (3)

[a] Caused by pressure on the contralateral side during surgery

[b] Includes pressure ulcers
